# Stacking Effects on the Optoelectronic Properties of 2D Perylene-Zn-Porphyrin-Based COFs

**DOI:** 10.1021/acs.jpcc.5c08341

**Published:** 2026-03-10

**Authors:** Valentin Diez-Cabanes, Sergio de-la-Huerta-Sainz, Elisabeth Escamilla, Pedro A. Marcos, Alfredo Bol-Arreba, Kathryn McCarthy, Roberto González-Gómez, Santiago Aparicio, Pau Farràs

**Affiliations:** † International Research Center in Critical Raw Materials for Advanced Industrial Technologies (ICCRAM), 16725 University of Burgos, 09001 Burgos, Spain; ‡ Department of Chemistry, University of Burgos, 09001 Burgos, Spain; § Department of Physics, University of Burgos, 09001 Burgos, Spain; ∥ School of Biological and Chemical Sciences, Ryan Institute, 8799 University of Galway, Galway, Ireland H91 TK33

## Abstract

Crystalline porous materials, such as covalent organic frameworks (COFs), have emerged as promising candidates for photocatalytic and optoelectronic applications due to their tunable architecture and capacity to mitigate charge recombination. The incorporation of highly aromatic organic building blocks that promote self-assembly and columnar growth enables the formation of COFs with a controlled layer thickness. However, the influence of interlayer stacking on the structural and optoelectronic behaviors of these materials remains poorly understood. In this work, we combine experimental and theoretical approaches to elucidate the stacking-induced evolution of perylene–Zn–porphyrin COFs. Spectroscopic and microscopic analyses, supported by density functional theory (DFT) calculations, reveal that self-assembly through AA stacking markedly modifies both the geometry and electronic structure. The transition from nonplanar 2D architectures to planar multilayered frameworks results in reduced band gaps, inversion of the frontier crystalline orbital delocalization, and a shift of absorption dominance toward the porphyrin units. These findings demonstrate that controlled layer stacking is a viable strategy to tailor the electronic and optical properties of stacked 2D COFs, paving the way for their integration into high-performance optoelectronic devices.

## Graphical abstract

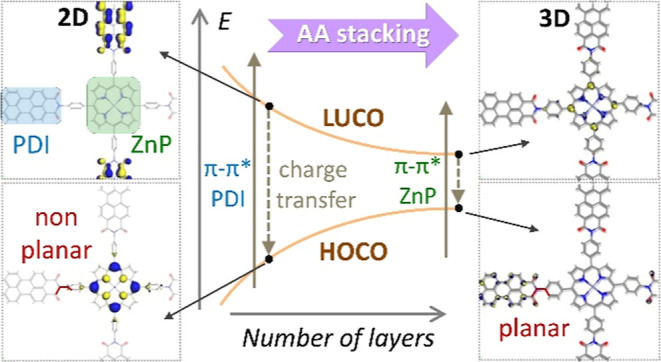

## 1 Introduction

Covalent organic frameworks (COFs), crystalline porous materials, are constructed by molecular building blocks through strong covalent bonds.^1^ Building blocks and linkers made of molecular units, such as triazine, imine, imide, olefin, or oxazole,^2^ as well as aromatic molecules that can control stacking^3^ and different topologies,^4^ have been considered. COFs exhibit inherent porosity arising from the void spaces between covalently linked organic building blocks. COF synthesis enables the formation of well-defined structures with atomic-level precision.^5^ Due to this, their porous architecture can be tailored by adjusting the size, shape, and chemical functionality of the building units, offering opportunities for several applications.^6^ The rational selection of structural building blocks enables the design of well-defined, task-specific COFs, suitable for diverse technologies, including environmental remediation,^7^ gas storage,^8^ CO_2_ capture and conversion^9^ electro- and photocatalysis,^9,10^ and drug delivery.^11^ On the same vein, the semiconducting nature of the COF organic components opens the door to their integration in a wide range of optoelectronic devices,^12^ including light-emitting diodes (LEDs),^13^ solar energy technologies,^14^ or supercapacitors.^15^

The diverse applications of COFs stem from their physicochemical properties, such as high thermal stability, low densities,^16^ porosity, and large specific surface areas.^17^ Stiffness is also an important characteristic that can be considered for designing 2D^18^ or 3D^19^ COFs. In this regard, extended π-conjugated building blocks, such as perylene, promote the separation and migration of carriers, significantly improving the crystallinity and photoelectrical COF properties.^20,21^ In the same vein, Zn-porphyrin building blocks also present several suitable properties such as strong visible light harvesting, efficient exciton transport, resistance to aggregation, enhanced electron transfer, and redox activity.^22,23^ What is more, the utilization of building units such as perylene^24,25^ and porphyrin^26^ in the development of 2D COFs is driven by their suitable low donor–acceptor (D–A) energy gaps, high carrier mobility, and thermal stability, which are crucial for maintaining the structural integrity of the framework.^27–29^ Recently, 2D COFs based on porphyrin and perylene units with benzene linkers were investigated,^30–32^ which showed an ultrafast charge carrier mechanism using both experimental and theoretical techniques.^32^ Stacking of 2D units to form layered COFs samples gives rise to the growth of columnar structures with controlled porosity. Due to the overlap of orbitals between the different layers, the multilayered 2D material presents a band-like charge transport^33^ in the stacking direction, thus leading to high conductivities, which are comparable to those achieved by standard inorganic semiconductors. Overall, self-assembly of COFs allows for the fine-tuning of their optoelectronic response^34^ via dimensional and quantum confinement effects^35^ or via stacking engineering.^36^ Moreover, one can combine COFs with a different number of layers to build a multidimensional material with the enhanced properties of each particular component, in a similar manner to other inorganic materials.^37^ Despite several works having been dedicated to the study of the conductivity in columnar 2D COF structures,^38^ a complete picture of the self-assembly process, and how the stacking process impacts the optoelectronic response of the 2D COFs is still lacking.

In this context, we conducted a combined theoretical and experimental study to shed light on the main structural, optoelectronic, and thermodynamic properties of multilayered COFs. In addition, the self-assembly process has been tracked by analyzing the evolution of qualitative and quantitative changes in the vibrational properties as a function of the number of COF layers. To illustrate this methodology, a light-harvesting COF was chosen to illustrate this methodology, which was constructed from 10,15,20-tetrakis­(4-aminophenyl)­porphyrin (TAPP) and perylene-3,4,9,10-tetracarboxylic perylene (PTCDA) building blocks, followed by postsynthetic incorporation of Zn into the porphyrin macrocycle, thus creating a perylene di-imide (PDI)-Zn porphyrin-(ZnP) COF (see Figure 1). First, the characterization of the crystalline structure and optoelectronic properties of the PDI-ZnP-COF material was assessed via vibrational spectroscopy, microscopy, and theoretical modeling performed in a monolayer model. The evolution of the optoelectronic (i.e., electronic structure and excited state properties) and structural [X-ray diffraction (XRD) patterns, vibrational spectra] properties with increasing number of stacked monolayers was further investigated via periodic time-dependent density functional theory (TD-DFT) calculations. Note that, despite the TD-DFT approach, or other perturbative techniques, such as the combined GW approximation and Bethe Salpeter equation (GW/BSE),^39^ having already been employed to estimate the excited state properties of periodic 3D or monolayer COFs,^40–42^ this work represents the first time that this methodology has been implemented in multilayered frameworks. As a matter of fact, this computational approach allowed us to gain access to several properties that were directly contrasted against experimental measurements, such as the interlayer distance of the multilayered COF or the absorption spectra. Our multiscale modeling approach ranged from monolayer models investigated at the hybrid functional density functional theory (DFT) level up to the multilayer models where, due to the high computational cost derived from the increase of the number of atoms considered (up to 1100), the DFT-based tight binding (DFTB) technique was used instead. A similar approach has been successfully applied to investigate the structural,^43^ mechanical,^44^ and stacking motifs^45^ of COFs presenting large unit cells (>200 atoms). This machinery enabled us to fairly reproduce realistic structures, thus having access to relevant structural data (i.e., interlayer distances), and, what is more, it can be easily extrapolated to the study of other complex porous materials.

**Figure 1:**
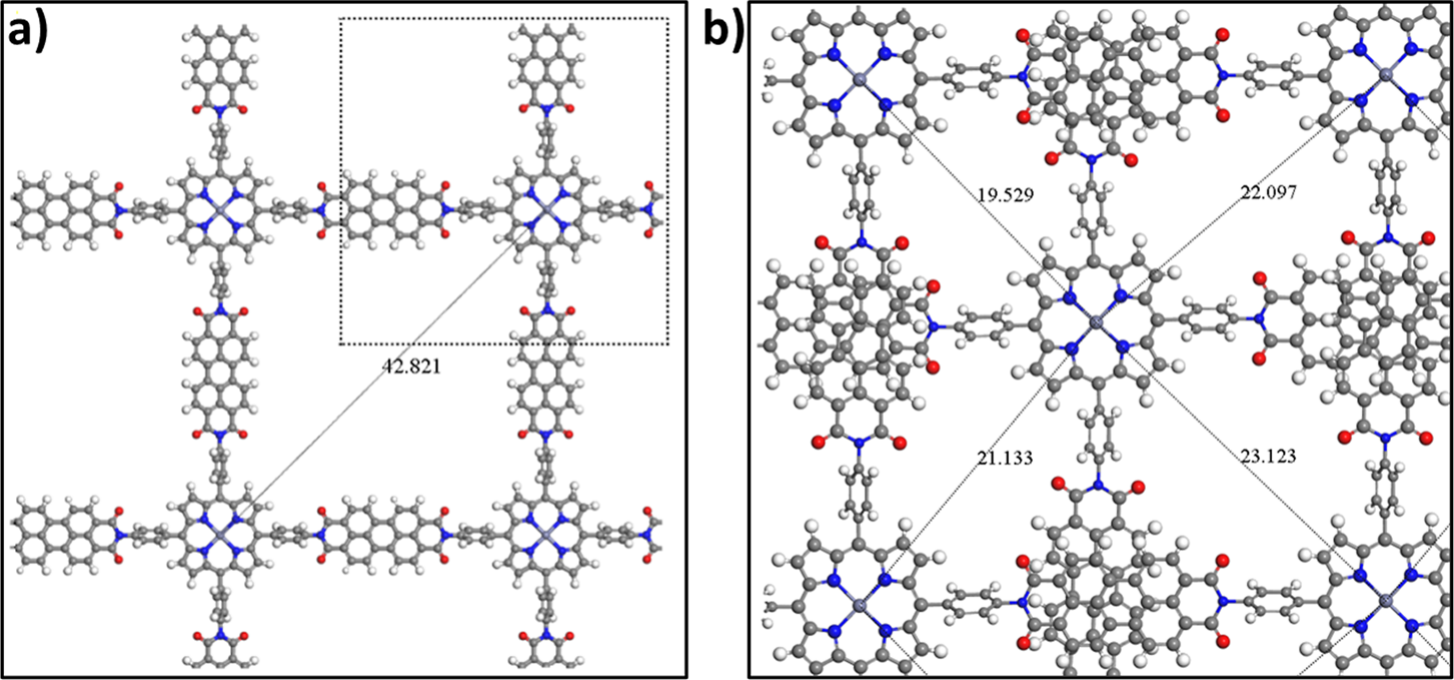
Top views in (001) plane of (a) the PDI-ZnP-COF monolayer structure and 2 × 2 × 1 supercell, which is indeed representative of the AA stacking; and of the (b) AB stacked PDI-ZnP-COF monolayers. Dashed line indicates the unit cell.

## 2 Methodology

### 2.1 Synthetic Procedures

#### 2.1.1 Synthesis of 5,10,15,20-tetrakis­(4-aminophenyl)­porphyrin

The porphyrin-based building block, TAPP, was synthesized in two steps, using a modified literature procedure^46^ as follows.

##### 2.1.1.1 Synthesis of 5,10,15,20-tetrakis­(4-nitrophenyl)­porphyrin

4-nitrobenzaldehyde (11.0 g, 0.073 mol) and acetic anhydride (12.0 mL) were dissolved in propionic acid (300 mL) in a round-bottom flask and stirred vigorously. The solution was brought to reflux, and then freshly distilled pyrrole (5.0 mL, 0.072 mol) was added dropwise. The reaction mixture was refluxed for an additional 30 min and then cooled to room temperature. A dark blue precipitate was collected by filtration, washed with a large quantity of cold water and methanol, and dried in an oven at 110 °C for 2 h. The dried dark purple powder was dissolved in pyridine (80 mL) and refluxed for 1 h. Once cooled, the precipitate was filtered and washed with cold acetone, giving purple crystals in ca. 5% yield. ^1^H nuclear magnetic resonance (NMR) (500 MHz, CDCl_3_), δ (ppm): 8.82 (m, 8H), 8.68 (m, 8H), 8.40 (m, 8H), −2.82 (s, 2H).

##### 2.1.1.2 Synthesis of TAPP

Previously synthesized 5,10,15,20-tetrakis­(4-nitrophenyl)­porphyrin (2.0 g, 0.003 mol) was dissolved in 12 M HCl (200 mL) in a round-bottom flask and heated to 70 °C while stirring. Once completely dissolved, SnCl_2_·2H_2_O (9.0 g, 0.040 mol) was added slowly to the solution and stirred for 30 min. The reaction mixture was cooled to room temperature, placed in an ice bath, and neutralized with 35% NH_3_. The product was filtered, dissolved in acetone, and then filtered again through silica. The solvent was removed by rotary evaporation to give a purple powder, which was then dried further under high vacuum to produce TAPP in ca. 25% yield. ^1^H NMR (500 MHz, CDCl_3_), δ (ppm): 8.90 (s, 8H), 8.00 (d, 8H), 7.10 (d, 8H), 4.00 (s, 8H), −2.72 (s, 2H).

#### 2.1.2 Synthesis of Perylene Diimide-Porphyrin (PDI-P) COF

Perylene-3,4,9,10-tetracarboxylic dianhydride (PTCDA; 0.044 g, 0.110 mmol), Zn­(OAc)_2_ (0.250 g, 1.360 mmol), and 5.0 mL of degassed dimethylformamide (DMF) were added to a Teflon liner under inert conditions. The mixture was sonicated for 20 min, and then 0.1 mL of 12 M HCl, TAPP (0.050 g, 0.074 mmol), and 5.0 mL of additional degassed DMF were added to the reaction mixture under a continuous nitrogen flux. The Teflon liner was placed in a stainless-steel autoclave and heated in an oven at 160 °C for 18 h. Once cooled, the resulting insoluble solid was collected using vacuum filtration and washed with copious amounts of DMF, water, methanol, and dichloromethane. The product was dried at 110 °C to yield 25 mg of PDI-P COF as a dark brown powder.

#### 2.1.3 Synthesis of Zn Metalated PDI-P (PDI-ZnP) COF

PDI-P COF (30 mg) was dispersed in 30 mL of chloroform in a 100 mL round-bottom flask. Separately, Zn­(OAc)_2_ (20 mg, 0.11 mmol) was dissolved in 15 mL of methanol and then added to the COF dispersion. The mixture was refluxed for 18 h, and once cooled, the COF was collected by filtration. The product, PDI-ZnP-COF, was washed with water and ethanol, and then dried under vacuum. EDX elemental analysis of metalated material: Zn 5.55%.

### 2.2 Materials Characterization

NMR spectra were recorded using a Varian VNMRS 500 MHz 54 mm AR spectrometer and processed with Bruker Topspin software calibrated against solvent peaks according to published values. Fourier transform infrared (FTIR) spectra were obtained on a PerkinElmer Frontier FTIR; powders were diluted in CsI pellets prior to recording. Ultraviolet–visible (UV–vis) spectrometry was carried out on a Cary 5000 UV–vis–NIR spectrometer (200–2500 nm range) with a deuterium UV lamp light source using R928PTM (UV–vis) or polytetrafluoroethylene (diffuse reflectance spectroscopy (DRS)) detectors using a xenon lamp. Liquid-state measurements were recorded in a quartz cuvette with a path length of 10 × 10 mm; samples were dispersed in either heptanoic acid or ethanol for 30 min before analysis. Solid-state measurements were performed by using a diffuse reflectance accessory (DRA), and pure MgO was used as a blank reference. Powder X-ray diffraction (PXRD) measurements were carried out on a Bruker D2 Phaser instrument operating using a Cu Kα (λ = 1.54178 Å) radiation source and a Lynxeye detector at room temperature, with samples mounted on a zero-background silicon single-crystal sample stage. Microscopy measurements were performed in the “center for microscopy and imaging” at Galway University of Galway. Scanning electron microscopy-energy-dispersive X-ray analysis (SEM–EDX) measurements were conducted on a Hitachi S-4700 SEM instrument with an EDX spectrometer. Samples were dispersed in ethanol via 3 h sonication, then drop-cast and dried on clean silicon wafers (5 × 5 mm), and coated with gold prior to imaging. Transmission electron microscopy (TEM) measurements were carried out on a Hitachi H7500 electron microscope; images were taken at 100 kV and room temperature and are shown in their raw data form. Samples were exfoliated by sonication in ethanol for 3 h or heptanoic acid for 1 h before being drop-casted on 200 mesh copper grids with formvar/carbon membrane coatings; grids were dried under vacuum for 8 h before imaging. Excitation and emission spectra were recorded on a Cary Eclipse fluorescence spectrophotometer using quartz cuvettes with a 10 × 10 mm path length; samples were sonicated for 30 min in degassed heptanoic acid prior to analysis.

### 2.3 Materials Modeling

#### 2.3.1 Geometry Optimization and Electronic Structure

##### 2.3.1.1 Computational Details

The smallest system analyzed is a periodic 2D monolayer COF in which the unit cell contains 149 atoms. This system, along with the stacked material, is described within DFT approximation using the Dmol3 code implemented in the Materials Studio Suite.^47^ In the case of the monolayer system, wave functions are constructed employing a DNP (double numerical basis set with polarization) basis set (double-ζ basis set augmented with polarization functions) using the Generalized Gradient Approximation (GGA) with the Perdew–Burke–Ernzerhof (PBE) exchange correlation functional.^48^ Additionally, semicore pseudopotentials (DSPP) were applied with an energy accuracy threshold cutoff of 0.1 eV atom. To validate the results obtained for multistacked larger systems with a DFT-TB method, the local density approximation (LDA) with the Perdew–Wang–Ceperley (PWC) exchange–correlation functional was used, also incorporating DSPP employing the same orbital cutoff quality as in the GGA calculations. All simulations were conducted at 0 K, with a convergence criterion for the self-consistent field set at 10^–4^ eV.

Since increasing the number of atoms in molecular simulations is a critical issue, large systems often require the use of less computationally demanding approaches. These methods must adequately describe chemical processes with predictive capabilities comparable to those of higher computational cost techniques, such as DFT at the GGA theoretical level, typically used for smaller systems. Indeed, to simulate the stacking effect, larger unit cells containing 1110 atoms were required. Therefore, DFT-TB simulations were employed to mimic the atomic interactions by utilizing the Slater–Koster set from the 3ob library,^49^ as implemented in the Biovia Materials Studio suite. The tolerance for self-consistency in these calculations was also set to 10^–4^ eV. To account for weak interactions, the Lennard–Jones potential was applied to describe the dispersion correction.^50^ To validate the capacity of the DFT-TB technique for addressing a problem involving such a large number of atoms, as a consequence of the PDI-ZnP COF stacking process, we have compared the properties calculated by DFT-TB with those computed by different types of DFT approximations: LDA and GGA. In detail, we have computed two representative structural and electronic property descriptors: the energy band gap and the IR frequency associated with the imide groups, which are collected in Table S1. The results shown in this table indicate that the values computed with the three methodologies are quite similar, with DFT-TB being slightly closer to the GGA method. This confirms the suitability of DFT-TB approximation to properly capture the electronic structure of the multilayered structure by relying on lower computational demanding semiempirical methods. As a matter of fact, this methodology has been previously and successfully used to describe the structural and electronic features of similar COF structures.^51,52^

##### 2.3.1.2 Theoretical COF Cell Models

The initial unit cell of the periodic system, as shown in Figure 1, was constructed by using three structural components: Zn-porphyrin macrocycles, perylene, and benzene linkers. The Zn-porphyrin macrocycles are positioned at the nodes of the framework, with the Zn atom 4-fold coordinated with the nitrogen atoms of the porphyrin, occupying the central position of a planar square conformation, forming a chelate-like structure. Benzene molecules act as linkers between the Zn-porphyrin rings and the perylene moieties. This configuration is well-known for promoting the delocalization of the Zn atom’s electronic cloud^53^ throughout the π-electrons system, thereby enhancing its light harvesting capacity.

The 2D-COF unit cell, delimited by a dashed line in Figure 1, comprises a Zn-porphyrin macrocycle connected to two segments of pristine PDI by a benzene molecule. This unit cell contains 149 atoms and exhibits a tetragonal crystal structure (*a* = 30.42 Å, *b* = 30.25 Å, *c* = 10 Å; γ = β = α = 90°), as represented in Figure 1. Both the COF motif and the supercell were fully relaxed considering periodic boundary conditions (PBC) and a vacuum top layer of 40 Å to avoid the interaction between neighboring cells. Then, to assist in the experimental characterization of the material, infrared (IR) and XRD spectra of the relaxed structures were calculated using the frequencies and reflex modules of the Biovia Materials Studio software.

#### 2.3.2 Optoelectronic Properties

For a more accurate representation of the COF electronic structure, the ground state properties of the optimized PDI-ZnP-COF cells were calculated at the DFT level by employing both standard (PBE) and hybrid (PBE0)^54^ functionals with an electron density energy cutoff equal to 600 Ry. Then, the analysis of the COF optical properties was pursued by computing their vertical excitations by means of the time-dependent density functional perturbation theory (TD-DFPT) linear response approach,^55^ within an energy cut off of 200 Ry for the excited state electronic density, and an energy convergence threshold of 10^–5^ eV. It is important to highlight that both standard and hybrid functionals yielded to similar composition of the PDI-ZnP-COF band edges, as it can be observed in the projected density of states (PDOS) represented in Figures S1 and 4a computed with PBE and PBE0, respectively. Therefore, both functionals should show, in principle, an identical nature of the lowest energy states, which justifies the choice of PBE functional for studying the COF optical properties. Valence and core electrons were accounted for by using a double-zeta valence polarized (DZVP) MOLOPT basis set^56^ and Goedecker–Teter–Hutter (GTH) pseudopotentials,^57^ respectively. Notably, similar theoretical approach has been recently implemented to investigate the photophysics of similar hybrid porous materials.^58,59^ The cell dimensions in the *z*-direction exceeded 50 Å in order to avoid the interaction between neighboring COFs. Note that due to their high computational cost of these calculations, only optoelectronic calculations of multilayered systems up to three layers (L1, L2, and L3) were considered. All calculations related with the optoelectronic property characterization of PDI-ZnP-COF s were carried out at the Γ point within the CP2K package.^60^

## 3 Results and Discussion

### 3.1 In Silico and Experimental Material Characterization

#### 3.1.1 Structural and Thermodynamic Properties

PDI-P COF was synthesized by a condensation reaction between 5,10,15,20-tetrakis­(4-aminophenyl)­porphyrin (TAPP) and perylene-3,4,9,10-tetracarboxylic dianhydride (PTCDA) under inert solvothermal conditions in DMF, using zinc acetate and HCl as catalysts. The material was then metalated with Zn (II) to give the final product PDI-ZnP-COF. The formation of the network was confirmed by Fourier transform IR (FTIR) spectroscopy, focusing on the signals attributed to the di-imide bond; vibration peaks of the imide carbonyl groups ν (C=O) were observed at 1695 cm^–1^ and 1660 cm^–1^, along with a peak at 1360 cm^–1^ ν (C–N), which indicates a new C–N–C bond (Figure 2a top). Further characterization was performed with powder XRD (PXRD), which exhibits relatively intense peaks at 6.5°, 8.2°, 11.9°, 24.2°, and 27.5°, indicating a certain degree of long-range molecular order in the overall material. The diffraction peak at 27.5° is attributed to the π–π stacking interactions of the framework, as observed in other similar materials;^61^ d-spacing calculations showed a 3.2 Å separation between 2D PDI-P COF layers (Figure 2b top). Both techniques corroborated the formation of the COF, giving structural information about the bonding nature and molecular arrangement.

**Figure 2:**
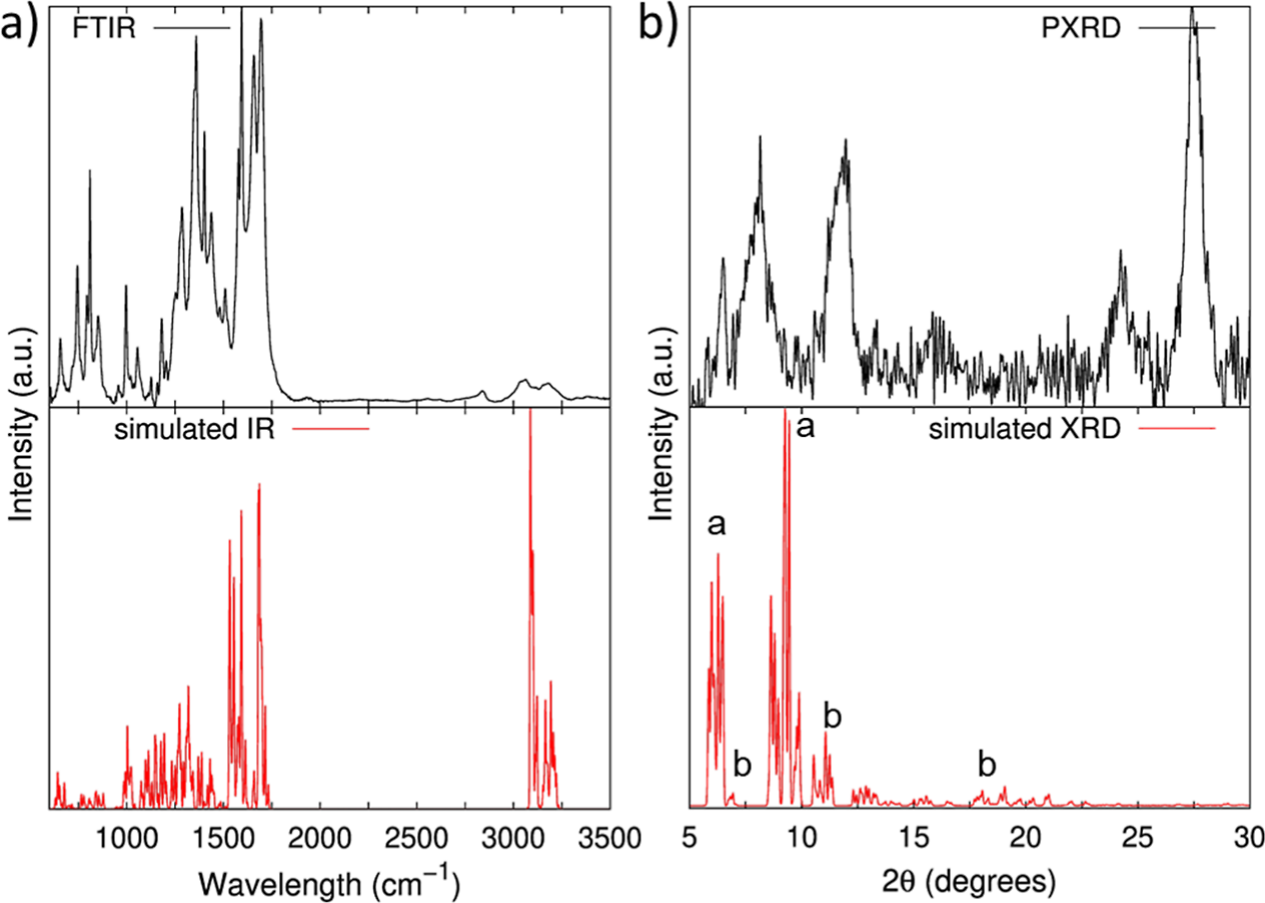
Vibrational and structural characterization of the PDI-ZnP-COF: (a) IR spectrum acquired experimentally via Fourier transform (FTIR) (top) and simulated (bottom panel); XRD diffraction patterns obtained experimentally via PXRD measurements (top) and calculated from the optimized structure of the PDI-ZnP-COF monolayer (bottom panel). Labels “a” and “b” in Figure 2b bottom stand for XRD patterns assigned to ZnP and PDI units, respectively.

SEM imaging was then performed on PDI-ZnP-COF to study the morphology of the framework, showing a densely aggregated material with irregularly shaped clusters, ranging in size from 0.1 to 2.0 μm (Figure S2). Optical and electronic properties are known to be affected when polymeric materials such as COFs are aggregated;^62^ therefore, exfoliation with a polar solvent and a high-boiling-point fatty acid, namely, ethanol and heptanoic acid, was carried out. Transmission electron microscopy (TEM) of the COF exfoliated in ethanol displayed heterogeneous structures with low dispersity along the grid, with sizes between ca. 40 to 900 nm; higher electronic density areas were localized, still showing the presence of aggregates. On the other hand, when the COF was exfoliated in heptanoic acid, a dispersing stabilizing agent,^63^ the aggregates were broken down into crystallites with spherical form and a mean size of 12 ± 3 nm (Figure S3).

In a subsequent step, with the aim of getting a deeper picture of the 2D-COF structure and rationalizing the geometrical features observed experimentally, we conducted a series of DFT calculations on the PDI-ZnP-COF monolayer. The optimized cell parameters (*a* = 30.4 Å and *b* = 30.3 Å) were very close to the experimental ones^32^ (*a* = *b* = 30 Å), where Zn porphyrins are conforming a perfect square network, as can be seen in Figure 1. In the porphyrin ring, the average bond length *d*(Zn–N) between Zn and the N atoms is 2.06 Å, which is consistent with previous experimental and theoretical values.^64^ In Figure 1, it can be observed that the benzene ring linker and PDI are slightly twisted, with respect to the plane defined by the Zn–P moieties. The dihedral angles between perylene-porphyrin planes and between benzene linkers-porphyrin planes are approximately 30° and 60°, respectively. This out-of-plane displacement is attributed to the steric hindrance caused by the contact between hydrogen atoms belonging to porphyrin and benzene molecules (Figure 1). Thus, the torsion induced by benzene linkers may lead to electronic decoupling between PDI and ZnP moieties, as has been observed in previous works.^32,65,66^ Nonetheless, this torsional feature in the single monolayer is not preserved when moving to the multistacked sample, as will be discussed in Section 3.2.1.

The calculated IR spectrum of the optimized PDI-ZnP-COF monolayer is shown in Figure 2a bottom. The most significant vibrational modes from the computed IR spectrum are reported in Table 1. The IR spectrum exhibits several broad and intense bands, which can tentatively be attributed to the individual moieties: benzene linker, PDI, and porphyrin. The analysis of the resulting frequencies reveals different natures of vibrational modes. The bending mode of the aromatic linker has a frequency of 209 cm^–1^. On the other hand, the frequency of 1151 cm^–1^ is associated with a bending mode between perylene and the benzene linker. Additionally, different frequencies corresponding to the bending mode of the C–H bond have been obtained. The 1162 and 1181 cm^–1^ frequency modes are attributed to the perylene, whereas the one at 1173 cm^–1^ is associated with the linker. Concerning the stretching modes, the lowest frequency region corresponds to ν­(C–C) of the porphyrin, with bands appearing within the range 1500–1600 cm^–1^. The ν­(C=O) band, corresponding to the carbonyl group, is observed at frequencies in the 1697–1728 cm^–1^ range. These values are consistent with the experimental results obtained in this investigation and previous theoretical and experimental studies performed in similar porphyrin COF.^32,67^ A vibrational mode associated with the N atom in the imide region, ν­(C–N), occurs at 1139 cm^–1^ and is coupled to the linker structure. This value is blue-shifted with respect to experimental frequencies from Figure 2a, top, and those from the existing literature.^24^ As will be discussed with more detail in Section 3.2.1, this discrepancy arises from the lack of interlayer interactions constraining the motion of these atoms, which are not present in the monolayer model discussed here. Additionally, the asymmetric and symmetric modes of porphyrin ν­(C–H) were found at 3121 and 3143 cm^–1^, respectively. These values are comparable with both the available experimental and theoretical data for the Zn-porphyrin moiety, as shown in Table 1.^64,68^ Furthermore, several coupled vibrations are observed due to oscillation in the porphyrin macrocycle. The type of substituent also affects the IR spectral frequencies, as previously reported.^64^

**Table 1:** IR-Calculated and Experimental Frequencies (cm^–1^) of the Main Vibration Modes of the 2D PDI-ZnP-COF Monolayer^a^

mode	calculated	FTIR spectrum	literature
δ(C3H6)	209		
δ(perylene-C6H4)	1151		
δ(C–H) perylene	1162, 1181		
δ(C–H) CH	1173		
υ(C–N–C) imide	1139	1359	1340–1360 ^69^
υ(C–C) porphyrin^b^	1500–1600	1577, 1595	1470–1605 ^70^
υ(C=O)	1697–1728	1695	1695,^71^ 1705 ^72^
υ(C–H) porphyrin	3121^as^, 3143^s^	3065, 3180	3037,^68^ 3204 ^64^

a Symmetric (s) and Asymmetric (as) Modes

b Coupled vibration.

Afterward, the phonon structure of the material was computed to estimate their thermodynamic properties, including free energy, entropy, and enthalpy. The thermodynamic characteristics up to 1000 K for the considered PDI-ZnP-COF monolayer are reported in Figure S4. It has been observed that the heat capacity as a function of temperature verifies Debye’s law at low temperatures (*T* < 400 K) and approaches the Dulong–Petit’s limit at higher temperatures, when exceeding the Debye’s temperature. This behavior is a consequence of the fact that anharmonic effects predominate and cannot be captured by vibrational calculations relying on the harmonic approximation. The noticeable increase in enthalpy with temperature indicates a significant internal energy gain, which is associated with a considerable thermal expansion of the material, implying changes in porosity upon heating. This behavior is corroborated by the strong increase in entropy with temperature, reflecting greater atomic mobility and a larger number of available microstates. The nonlinear decrease in free energy with increasing temperature results from the combined effects of rising atomic mobility, structural changes in the 2D material, and increasing volume. Therefore, material disruption and porosity changes upon heating should be expected for the PDI-ZnP-COF considered in this study, which, combined with its ability for thermal storage, suggests that this material could be considered a good candidate for outdoor technological applications exposed to large temperature variations.

To further characterize PDI-ZnP-COF, the XRD pattern was calculated, as shown in Figure 2b, bottom. The XRD pattern reveals the presence of two principal components: porphyrin and PDI. The porphyrin component exhibits main reflection peaks at 6.0° and 14.0°, 2θ units, labeled as “a” in Figure 2b bottom, comparing well with previous reported experimental and theoretical values for similar porphyrin COFs.^51,73^ The PDI component can be assigned to reflections at 6.3°, 8.6°, 11.1°, and 19.1°, peaks labeled as “b” in Figure 2b bottom. These values are close to the experimental reflections observed for DBP nanoplates, which were reported at 7.5°, 11°, 12.5°, 17.4°, and 20°.^74^ In general, these theoretical values show good qualitative agreement with the experimental peaks obtained in this study (see Figure 2b top), especially for the first three intense peaks located experimentally at 6.5°, 8.2°, and 11.9°, which fairly correspond to the signals found in the simulated XRD patterns at 6.3°, 8.6°, and 11.1°.

#### 3.1.2 Optoelectronic Properties

Optical properties were first evaluated experimentally for the material in bulk by solid-state ultraviolet–visible, observing broad absorption bands in the visible region at 408, 496, 538, and 609 nm (Figure S5), attributed to the presence of both photoresponsive ZnP and PDI fragments. Then, to evaluate the effects of the material aggregation on its properties, liquid-state UV–vis measurements expanded to the near-infrared were taken in both ethanol and heptanoic acid dispersions (Figure 3a). In both cases, an intense peak was seen at 445 nm, attributed to the π–π* transition (Soret peak) of the porphyrin unit,^75^ in addition to less intense bands at lower energy due to the presence of the perylene unit and the Q bands of the porphyrin; however, suspensions in heptanoic acid showed less defined transition bands and scattering effects due to the size of the crystallites. As a consequence, the PDI-ZnP-COF suspended in ethanol showed an additional band at 610 nm that cannot be recognized in the heptanoic acid dispersion. Also, absorption band intensities in relation to the Soret peak were higher for the sample dispersed in heptanoic acid, showing an extended homogeneous light absorption until 650 nm, whereas when dispersed in ethanol, light was absorbed primarily between 400 and 480 nm (Figure 3a). Overall, the marked differences observed between the UV–vis spectra recorded in ethanol and in heptanoic acid arise primarily from differences in the aggregation state and particle size distribution rather than from intrinsic changes in the electronic structure of the COF. Ethanol dispersions contain larger and more heterogeneous aggregates, as identified by SEM analysis, which enhance light scattering and suppress weaker electronic transitions, leading to spectra dominated by the porphyrin Soret band, whereas heptanoic acid acts as a dispersing and stabilizing agent, yielding smaller and more uniform crystallites, thereby reducing scattering effects and allowing lower-energy PDI- and charge-transfer-related absorption features to be more clearly observed. Due to the wider window of absorption of the PDI-ZnP-COF and better dispersibility, excitation and emission spectra were recorded in heptanoic acid suspension. Spectra showed a good degree of donor–acceptor overlapping, which allows energy transfer from the porphyrin unit to the perylene building block upon light irradiation (Figure S6).

**Figure 3:**
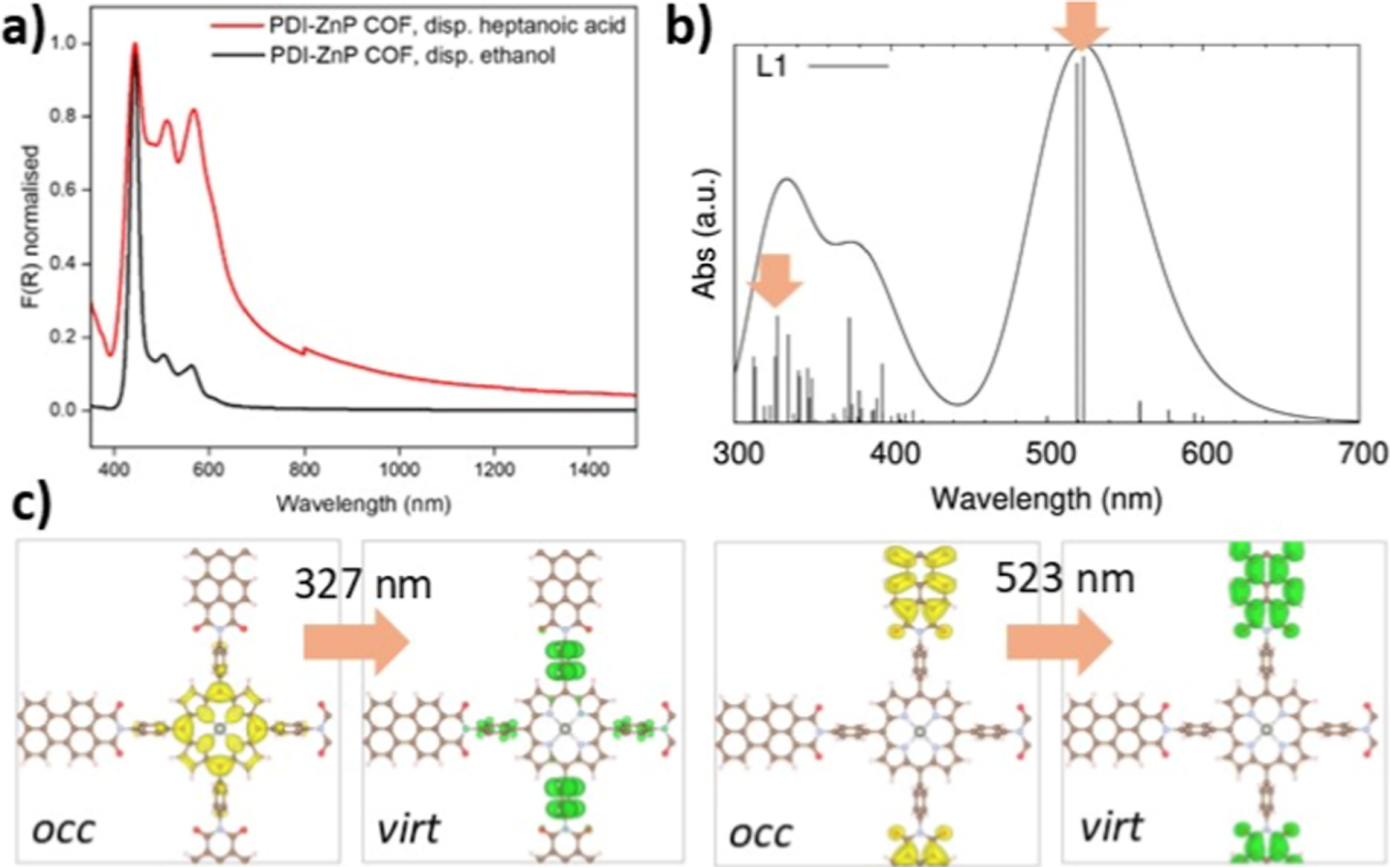
a) Liquid-state UV–vis spectra of PDI-ZnP-COF suspensions; ethanol (black) and heptanoic acid (red); (b) simulated TD-DFPT absorption spectra for the PDI-ZnP-COF monolayer where vertical bars represent the oscillator strengths of the states convoluting the spectra. The excited state properties of the main states belonging to this energy region are collected in Table S2; (c) lateral views of the shapes for the occupied (yellow) and virtual (green) crystalline orbitals involved in the main transitions of the excited states depicted with orange arrows in the simulated spectra. The top views for the rest of the crystalline orbital transitions composing the states collected in Table S2 are depicted in Figure S8. The iso-value used to plot the iso-surfaces was set to 0.02 au.

Then, with the objective of shedding light on the different photophysical phenomena underlying the absorption and emission features observed in the PDI-ZnP-COFs, we relied on the excited state properties estimated by TD-DFPT calculations. Note that the monolayer model was employed here as a reference system to disentangle intralayer electronic transitions from stacking-induced effects and to facilitate a direct analysis of the nature of the excited states. We first focused on the electronic structure of the optimized PDI-ZnP-COF monolayer (L1), which was analyzed by plotting the PDOS of the atoms composing the cell, as estimated by employing highly accurate hybrid functional DFT calculations (see Figure 4a). The main PDOS features are constituted by highest occupied/lowest unoccupied crystalline orbitals (HOCO/LUCO), which are separated from the rest of the higher-and lower-energy crystalline orbitals (COs) (i.e., H–1/L+1), as indicated in the plots. Then, to further characterize the composition of the frontier COs, we plotted the CO shapes corresponding to the H–1, HOCO, LUCO, and L+1, as represented in Figure 4b. Notably, our results indicated that while the HOCO is mainly localized on the porphyrin moiety, the LUCO is predominantly located in the PDI region (see Figure 4b), thus leading in principle to a marked ZnP-to-PDI charge transfer (CT) character for the band edge transitions, which hampers the recombination of the photogenerated charges. This behavior is consistent with the results reported through the optical properties evaluated in the experimental part carried out in this study and in other similar planar COF structures.^32^ Furthermore, the fact that LUCO is entirely localized on the PDI unit facilitates the charge transfer toward guest moieties within host–guest assemblies.^74^ Then, concerning the COs located further from the energy gap, while H–1 is localized on the PDI block, L+1 is mainly located in the ZnP moiety. Note that there is no CO delocalization along the different COF components due to the lack of planarity between the distinct building blocks.

**Figure 4:**
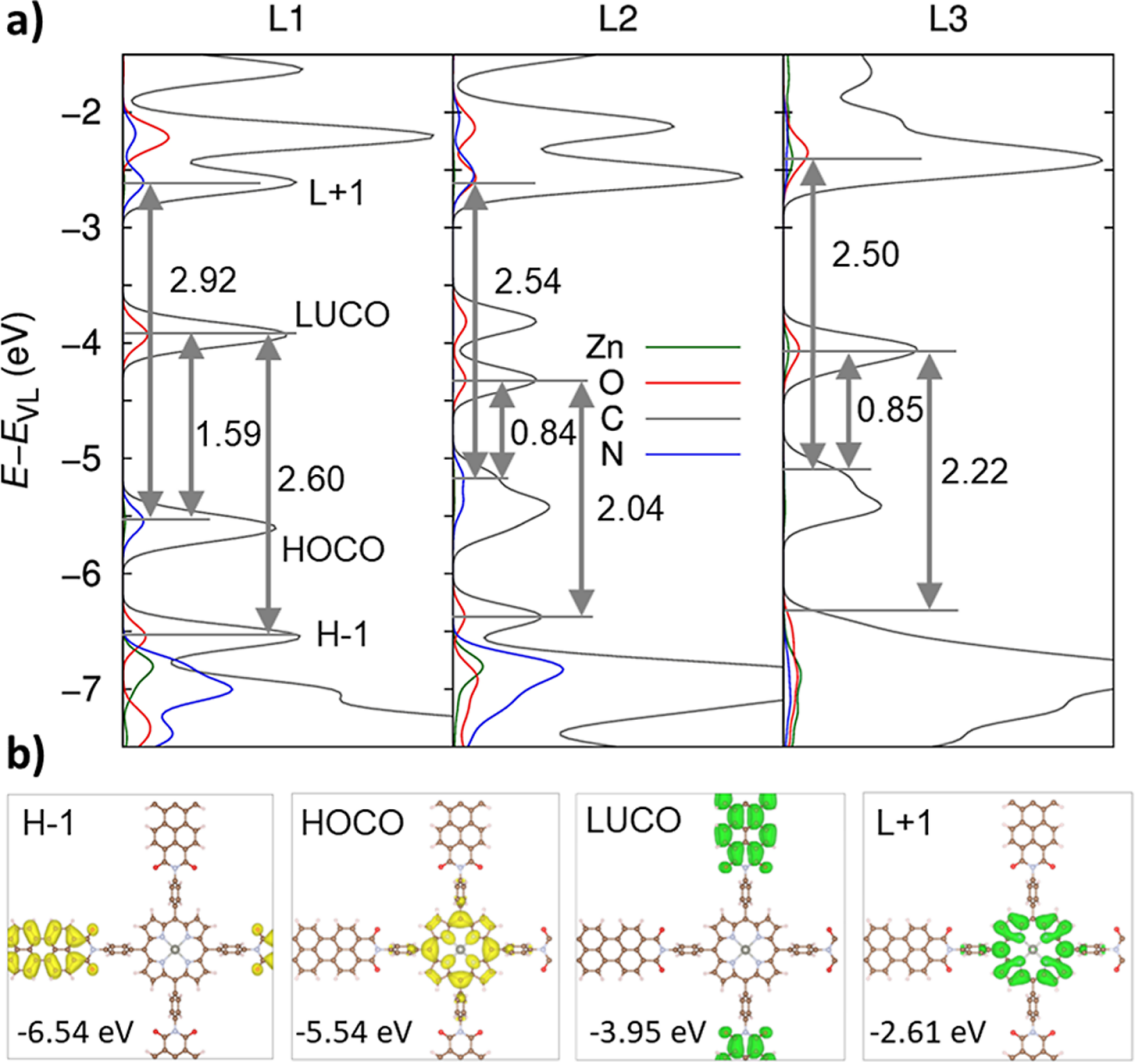
(a) PDOS of the atoms conforming the PDI-ZnP 2D COFs, owing to one (L1), two (L2), and three (L3) layers, as calculCOF monolayering hybrid DFT functionals (PBE0), where the vacuum level energy (*E*_VL_) was set as reference; and (b) top views of the shapes for the frontier crystalline orbitals (COs) indicated in the PDOS of the PDI-ZnP-COF monolayer. The frontier CO shapes for the rest of the PDI-ZnP-COF multilayered systems (L2 and L3) are collected in Figure S7. The iso-value used to plot the iso-surfaces was set to 0.02 au.

The resulting HOCO–LUCO gap was equal to 1.59 eV, which remains close to the optimal ones (i.e., 1.8–2.0 eV) for overall water splitting photocatalysis^76^ and is in reasonable agreement with the lowest energy signals from the emission spectra (Figure S6) appearing at 650 nm (1.91 eV). Concerning the energy gaps of the two COF components, in the case of PDI, it corresponded to the H–1 → LUCO gap with an estimated value of 2.60 eV, which is in good agreement with the PDI π–π lowest energy absorption bands located at 520 nm (2.38 eV);^77,78^ whereas for ZnP, it corresponded to the HOCO → L+1 gap with a value amounting to 2.92 eV, which goes in line with the porphyrin π–π* transition Soret peaks located at 445 nm (2.79 eV) observed in Figure 3a. However, this picture is given from a ground state perspective, so in order to properly address the electron–hole interactions induced upon excitation, we performed TD-DFPT calculations.

The simulated UV–vis spectrum of the PDI-ZnP-COF monolayer is depicted in Figure 3b, which shows remarkable qualitative agreement with the experimental spectrum represented in Figure 3a. Both spectra are dominated by three main absorption bands, the first one (located at 523 nm) being the most intense absorption. It is noteworthy to mention that the qualitative agreement between theory and experiment refers to the overall structure of the absorption spectrum and the assignment of dominant transitions to specific molecular subunits, rather than to a strict one-to-one correspondence of peak positions. Then, with the aim of discerning the nature of the excited states originating from these absorption bands, we plotted the principal COs involved in the main vertical transitions conforming the simulated spectra, which are represented in Figure 3c. Interestingly, the lowest energy bright band located at 523 nm is attributed to the PDI π → π* transitions, being again in perfect agreement with the PDI absorption bands observed at 520 nm in previous studies.^77,78^ In the experimental spectra, however, aggregation and stacking effects redistribute the oscillator strength, resulting in a dominance of porphyrin-derived transitions, particularly in the visible region. The higher energy bands centered at 373 and 323 nm are related to PDI and ZnP transitions, respectively, with a certain extent of delocalization along the phenyl linkers (see Figures 3c and S8). Nonetheless, these higher–energy transitions predicted below 400 nm in the simulations are weak and partially obscured in the experimental spectra due to scattering and baseline effects, especially in the heptanoic acid dispersions. Indeed, this picture is consistent with the energy gaps obtained from the electronic structure, since the lowest energy bright states of PDI are lower in energy with respect to those of ZnP. Finally, the dark excited states (owing oscillator strength 4–5 times lower with respect to the bright ones, see Table S2) located below in energy with respect to the first bright absorption band (λ > 600 nm) correspond to ZnP-to-PDI CT transitions (see Figure S8), which correlate with the absorption tails found experimentally at λ > 650 nm (see Figure 3a).

### 3.2 Stacking Effects of PDI-ZnP-COF Layers

#### 3.2.1 Structural Properties

Once we corroborated the suitability of the DFT-TB technique to reproduce the structural and optoelectronic properties of the PDI-ZnP-COF monolayer (see Section 2.3.1), we then investigated the impact of increasing the number of COF layers on those characteristics by adopting this approach. In the first step, the type of stacking and its impact on the pore size were investigated. Two PDI-ZnP-COF monolayers were arranged to compare the stability of the two most representative stacking patterns, AA and AB, as shown in Figure 1a,b, respectively. The AA configuration, where the components of the COF are perfectly aligned between the different layers overlapping each other, was found to be energetically more stable by 0.15 eV/atom. Regarding pore size, the diagonal distance between Zn atoms in the AA pattern is 42.70 Å, which is similar to that of the monolayer, whereas in the AB pattern, where one of the layers is shifted so that the porphyrin group is located in the center of the square hole formed by the other one, this size decreases, with main values ranging from 19.53 to 23.12 Å, as reported in Figure 1b. Additionally, the interlayer distance in the AA bistacked system is 3.92 Å, while in the less stable AB bistacked system, it is 3.38 Å. These behaviors and values are consistent with previous reports for porphyrin-based COFs, which noted an interlayer distance of 3.3 Å for AB stacking and 3.9 Å for AA stacking.^51^

The interaction mechanism between two layers in the same conformation (AA) is primarily due to the interaction of the π electrons in the vicinity of the perylene moieties, contributing to increasing the stability of this stacking arrangement. A fortiori, similar COF systems have shown agreement with the values here obtained in both experimental and theoretical studies, confirming AA patterns as the most energetically favorable stacking modes.^51,52,79^ These results strongly suggest the self-assembled nature of this COF material, a hypothesis supported by experimental evidence indicating that PDI-ZnP-COF building units, such as porphyrin and perylene, can form self-assembled structures.^80,81^

Once AA stacking was confirmed as the most stable configuration, we successively added more layers (up to 6) to the systems studied. Interestingly, the starting COF configurations are completely flat, but after the optimization, two different behaviors were obtained, both of which are highlighted in Figure 5. For the two- or three-stacked layers, the benzene linkers are rotated out of the original plane of the COF, increasing the distance between layers up to approximately 4 Å (Figure 5a). This behavior mimics the PDI-ZnP-COF monolayer in vacuum, wherein benzene ring linkers rotate, forming a dihedral angle with the plane defined by the porphyrin moiety of around 60° as well. However, unlike the case of the monolayer, the perylene moieties of the COF́s do not experience any rotation, remaining in the same plane as the porphyrin (upper inset in Figure 5a). This difference may be attributed to the large number of available degrees of freedom in the monolayer case, which progressively decreases as the number of layers increases because of the steric hindrances that are present, thus promoting the formation of a globally flat structure, as shown in the lower inset of Figure 5. As a consequence, this geometrical effect shrinks the interlayer COF distances from 4 Å in a two-layer system to 3.1 Å in the six-layer system, as illustrated in Figure 5b, which is in good agreement with our experimental results from XRD patterns (3.2 Å) and other previous studies reporting 3.3 Å.^32^

**Figure 5:**
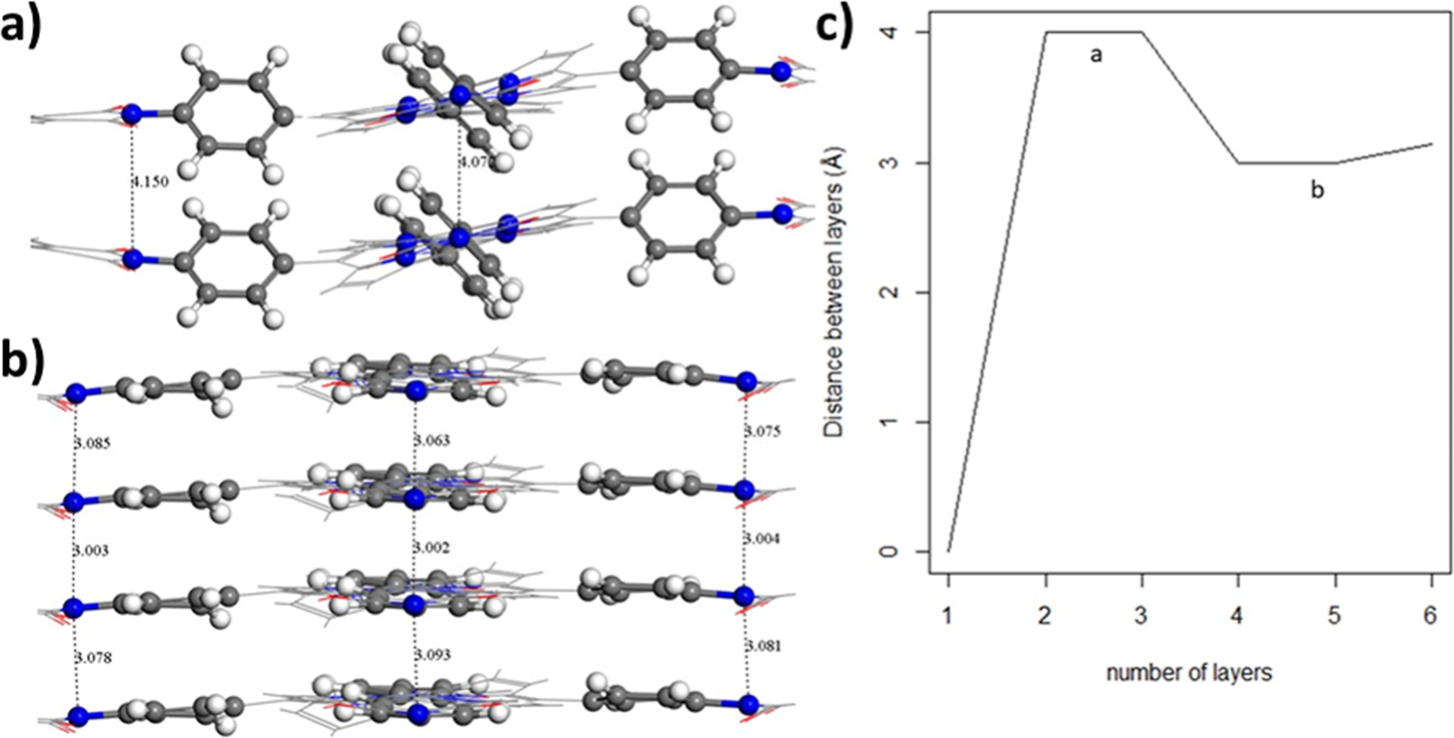
Stacking effects of the PDI-ZnP-COF: lateral view representing the prototypical stacking component orientation (a) from 1 to 3 layers and (b) from 4 to 6 layers (left panel); (c) evolution of the interlayer distance as a function of the number of layers (right panel). Note that labels “a” and “b” inside Figure 5c denote those interlayer distances for the COFs with *n* = 1–3 and *n* = 4–6 layers, respectively.

A distinctive indicator to confirm the formation of COF is the frequency associated with the stretching mode of the C–N–C linker, ν­(N–C). Theoretical calculations for a single monolayer indicate that the frequency most similar to the experimental evidence is 1134 cm^–1^, although it is associated with a bending mode rather than a stretching one. However, when the PDI-ZnP-COF exhibits a flat configuration upon stacking multiple layers, the numerical value shifts toward a true stretching imide mode, ν­(N–C) = 1341 cm^–1^, which is in line with our experimental signals located at 1360 cm^–1^ (see Figure 2a, top) and other mode frequencies reported previously^32^ at 1350 cm^–1^. As the number of layers increases, the frequency ν­(N–C) redshifts toward higher frequencies, approaching, asymptotically, the experimental value from 5 layers onward, as shown in Figure S9.

Afterward, the XRD patterns of the multilayered PDI-ZnP-COFs were also calculated. The X-ray diffractograms for the (Zn–COF)_*n*_ (*n* = 1–6), where *n* is the number of monolayers, are shown in Figure 6, whereas the mean peak positions are summarized in Table 2. The XRD patterns of the PDI-ZnP-COF monolayer are characterized by the presence of two principal components, namely, ZnP and PDI, as discussed in Section 3.1.1. However, upon increasing the number of stacked layers, two peaks at 6° and 7° associated with perylene become more prominent, together with a less intense peak at 9°, as can be observed in Figure 6. This behavior goes in line with the intense peaks located at 6.5° and 8.2° found in the PXRD patterns (see Figure 2b top).

**Figure 6:**
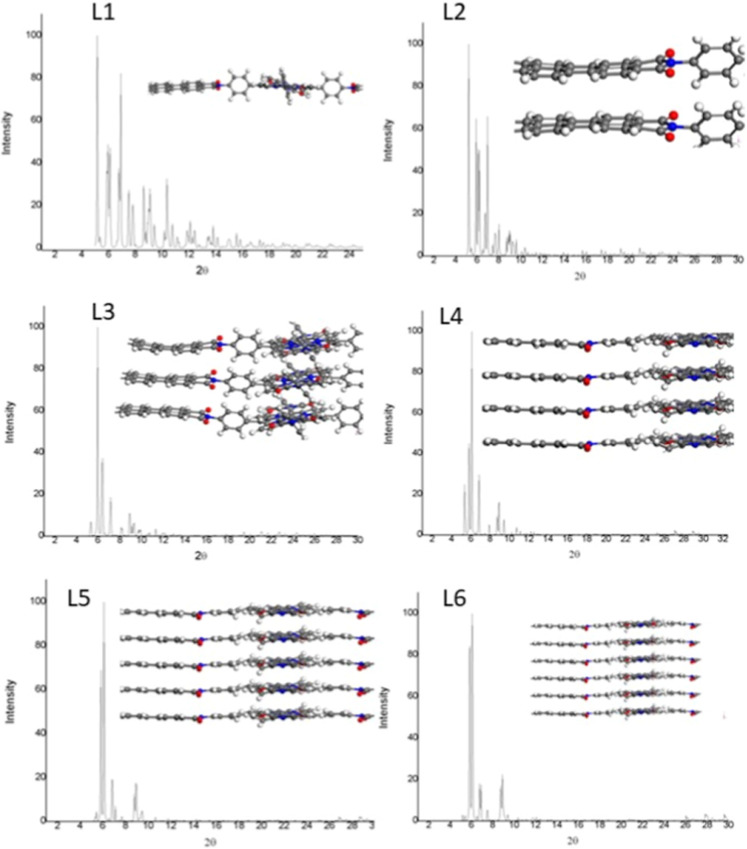
Calculated X-ray diffraction pattern for the optimized structures from 1 to 6 stacked PDI-ZnP-COF layers.

**Table 2:** Comparative Values between Several Stacked PDI-ZnP-COF Layers: HOCO and LUCO Orbitals and XRD Peaks

		HOCO	LUCO
number of COF layers	XRD peaks (2θ)	location	location
1	5.1^a^,5.9,6.9,8.7,10.5^a^,12.3,14.1	ZnP	PDI
2	5.3^a^,5.9^a^,6.9^a^,8.0	ZnP	PDI
3	5.4, 6.0^a^,6.5, 7.1^a^,9.0	ZnP	PDI
4	5.3,6.1^a^,6.9^a^, 9.0	PDI	PDI
5	6.1^a^,6.8, 9.0	PDI	ZnP
6	6.1^a^,6.8, 9.0	PDI	ZnP

a Intense peaks.

#### 3.2.2 Optoelectronic Properties

To explicitly account for interlayer interactions and aggregation effects, DFT and TD-DFPT simulations were additionally performed for bilayer and trilayer COF models, which represent minimal stacked systems capable of capturing interlayer electronic coupling at a comparable level of theory. As discussed below, these multilayer simulations provide a more realistic description of the experimental optical response and reveal qualitative changes in the absorption dominance upon stacking. We first focused on the electronic structure computed by hybrid DFT functionals for the smallest multilayered PDI-ZnP-COFs (*n* = 1–3, being *n* the number of layers). The PDOS of the atoms composing the optimized L1, L2, and L3 PDI-ZnP-COF cells are depicted in Figure 3a. Notably, the PDOS composition of the frontier COs from the PDI-ZnP-COF monolayer remains unaltered upon increasing the number of layers up to 3. Due to this, the shapes of the frontier COs for the multilayered L2 and L3 were found identical with respect to the monolayer (L1) ones, being HOCO/L+1 and H–1/LUCO localized on the ZnP and PDI moieties, respectively (see Figure S7). Since there is no planarity for L2 and L3 between the distinct COF components, the COs remained localized in a single building block. Nevertheless, the frontier CO energies were modified upon layer stacking due to their delocalization along the stacking direction: the HOCO levels were upshifted by 0.47 eV, whereas the LUCO levels were downshifted by 0.30 eV. Similar trends in the energy levels were observed by the COs located farther from the energy gap: an upshift of 0.21 eV for H–1 and a downshift of 0.04 eV for L+1. As a result, the HOCO-LUCO gaps were reduced by 0.74 eV due to the stacking effect, whereas the energy gaps associated with the PDI (H–1 → L) and ZnP (H → L+1) moieties were decreased by 0.38 and 0.52 eV, respectively. Nonetheless, due to the high computational load associated with hybrid DFT functional calculations, it was not possible to compute the electronic structure of multilayered PDI-ZnP-COFs with a number of layers larger than 3. To fill this gap, we computed the electronic structure of a complete series of stacked systems (L1-L6) by employing the DFT-TB technique. The CO shapes for all PDI-ZnP-COF multilayered systems are plotted in Figure 7a, whereas their spatial location along the COF building blocks is reported in Table 2. Strikingly, the location of these COs over the PDI-ZnP-COF́s building units varies with the progression of the stacking process, which correlates well with changes produced in interlayer distances. From 1 to 3 stacked layers, corresponding to longer interlayer distances­(4 Å), HOCO is predominantly located in the ZnP region, while LUCO is localized within the PDI units, which is fully consistent with the outcomes provided by more computationally costly hybrid DFT functionals. Consequently, as in the monolayer case, a CT process from the ZnP to the PDI is expected upon irradiation in the lowest energy region.^32^ When the number of stacked layers increases to 4, the interlayer distance decreases to 3 Å; this shrinking has a notable effect on the location of the frontier COs since both HOCO and LUCO are now located in the PDI zone. Lastly, from 5 layers onward, HOCO and LUCO exchange their positions with respect to the cases with fewer layers; i.e., HOCO moves to the PDI area, while LUCO is delocalized along the ZnP region. Due to this, the initial CT directionality of the lowest energy states is reversed when compared to the monolayer, pointing now toward the ZnP for L4-L6. Note that the DFT-TB parameters have been set to reproduce the pure DFT functional (i.e., GGA) energy gaps, which, in turn, usually underestimate the semiconductor energy gap around 30–40%.^82,83^ Then, with the objective of getting an accurate gap prediction of all set of multilayered COF systems, we used the perfectly linear correlation between the energy gaps computed at pure DFT vs hybrid DFT functional level for L1-L3 (see Figure S10) to be able to get energy gaps computed by the DFT-TB at the hybrid DFT functional level of accuracy. The evolution of these energy gaps with respect to the number of layers of PDI-ZnP-COFs is presented in Figure 7b. Indeed, the energy gap reduction observed previously for L1–L3 was further boosted by increasing the number of layers up to 6, going from 1.99 eV for the monolayer to 0.82 eV for L6, thus experiencing an energy gap decrease of 1.17 eV due to the stacking effect. The evolution of the gap and general geometric parameters can be related to the delocalization of the frontier orbitals, suggesting that from 5 layers onward, the contribution of the π-bonding electrons around the perylene becomes the most effective mechanism for charge transport.

**Figure 7:**
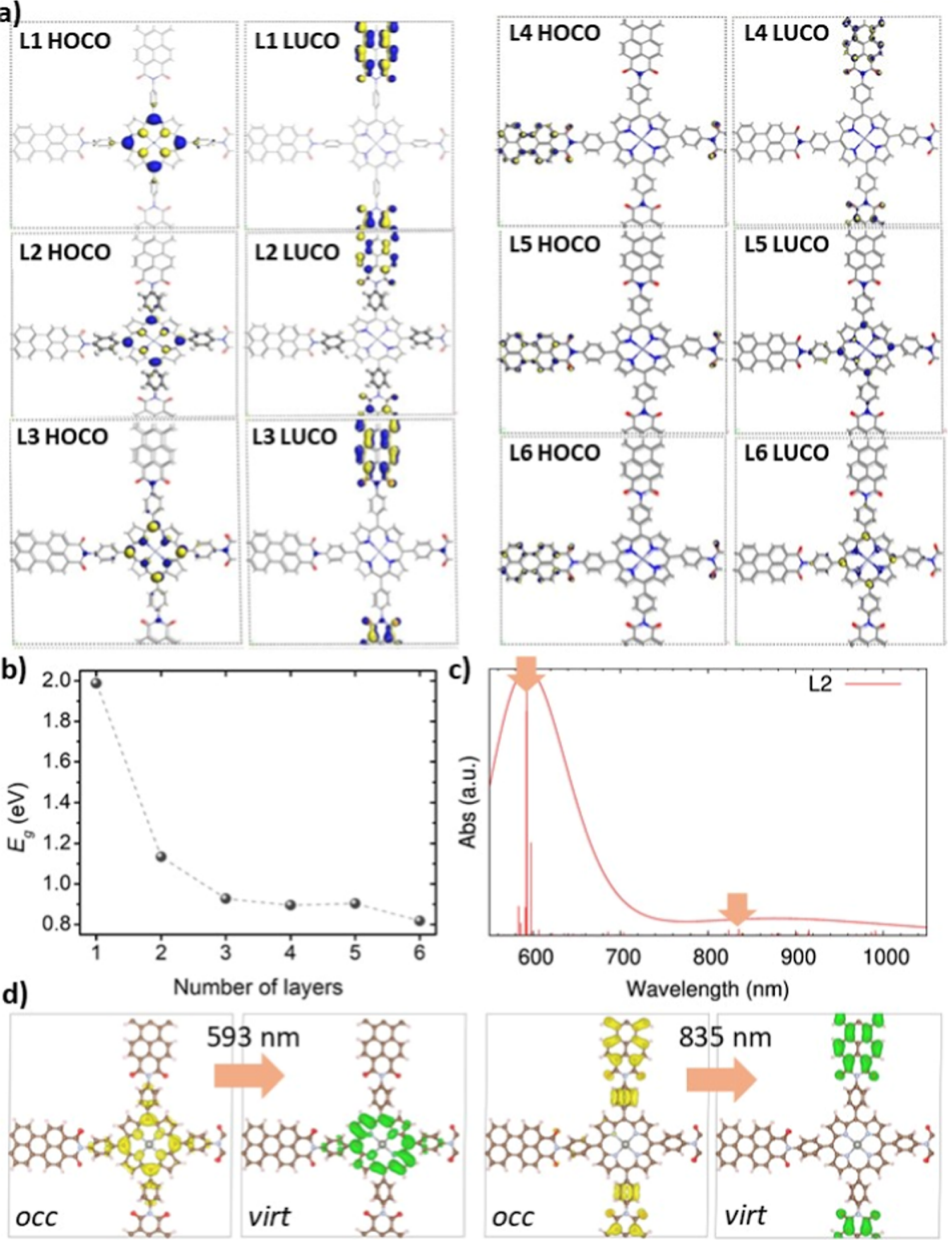
Evolution of the optoelectronic properties as a function of the number of layers (from 1 to 6) of the stacked PDI-ZnP-COF: (a) HOCO and LUCO shapes; and (b) corresponding energy gaps (*E*_g_) corrected with the correlation from Figure S10; (c) simulated TD-DFPT absorption spectra of L2 PDI-ZnP-COF where vertical bars represent the oscillator strengths of the states convoluting the spectra. The excited state properties of the main states belonging to this energy region are collected in Table S2; (d) lateral views of the shapes for the occupied (yellow) and virtual (green) crystalline orbitals involved in the main transitions of the excited states depicted with orange arrows in the simulated spectra. The top views for the rest of the crystalline orbital transitions composing the states collected in Table S2 are depicted in Figure S8. The iso-value used to plot the iso-surfaces was set to 0.02 au.

Finally, concerning the absorption characteristics of the multilayered PDI-ZnP-COFs, despite the TD-DFPT calculations of the COF monolayer attributing the most important absorption band in the lowest energy region to the PDI π → π* transitions, at the experimental level (see Figure 3a), the most intense absorption band is related to the ZnP moiety. In this regard, in order to get a closer representation of the absorption features of the PDI-ZnP-COF bulk material, we performed TD-DFPT calculations of the stacked PDI-ZnP-COFs containing 2 and 3 layers. The simulated spectra of the L2 PDI-ZnP-COF and the corresponding CO related to the most intense transitions from the spectra are presented in Figure 7c,d, respectively. In line with the picture provided by the electronic structure, where PDI energy gaps were about 0.5 eV lower with respect to the ones of ZnP, the lowest energy bright absorption bands corresponded to PDI π → π* transitions located at 835 nm (1.48 eV), followed in energy by the ZnP π → π* transitions located at 593 nm (2.09 eV). The underestimation of the computed ZnP Soret peak energies with respect to the experimental absorption spectra [445 nm (2.79 eV)] can be attributed to the use of the PBE functional (which usually underestimates the semiconductor gaps). However, this methodology is indeed required to compute the large number of states present in the lowest energy region of the multilayered COFs, being on the other hand, in good qualitative agreement with respect to higher level of theory approaches (i.e., hybrid functionals), as discussed in the methodological section (see Figure S1). Nonetheless, contrary to the COF monolayer (L1), for L2, the ZnP π → π* transitions become the dominant absorption bands of the lowest energy absorption region (displaying oscillation strengths 1 order of magnitude higher than the PDI π → π* transitions), thus being in perfect agreement with the experimental observables. Therefore, multilayer simulations demonstrate that stacking leads to a pronounced enhancement of porphyrin-dominated absorption bands, thereby reconciling the theoretical predictions with the experimentally observed intensity pattern. Then, the energy region below this absorption window (λ > 900 nm) is dominated by dark ZnP-to-PDI CT transitions with rather weak absorption (around 4 orders of magnitude lower with respect to the bright ZnP transitions). What is more, the band edge absorption region (∼1 eV below the first excited state) of L3 is also predominated by dark ZnP-to-PDI CT transitions, as can be observed in the COs depicted in Figure S8, right. Note that due to the large number of atoms containing L3, we focused on the nature of the first lowest excited states rather than on the overall shape of the simulated spectra, which indeed requires the computation of a larger number of states, thus becoming computationally prohibitive. Globally, these results highlight that relative absorption intensities in COF systems are highly sensitive to both structural aggregation and interlayer stacking, emphasizing the need to consider multilayer models when interpreting experimental optical spectra. The combined experimental and theoretical analysis, therefore, demonstrates that stacking is a key parameter controlling not only the energy of optical transitions but also their relative oscillator strengths.

To sum up, despite the present study focusing on a PDI–ZnP-based COF, the mechanisms identified here are expected to be broadly applicable to other two-dimensional COFs constructed from extended π-conjugated aromatic building blocks (i.e., porphyrin, perylene, pyrene, or phthalocyanine) exhibiting columnar stacking, although the quantitative details will depend on the chemical nature of the linkers and chromophores.

## 4 Conclusions

Herein, a joint theoretical and experimental study of a novel Zn-porphyrin-perylene-based COF displaying stacked structures due to columnar growth was carried out. Upon careful validation of our theoretical models against the experimental vibrational, XRD, microscopy, and absorption measurements, a step-by-step procedure has been adopted to elucidate the evolution of geometrical and optoelectronic properties from the monolayer system to the multistacked one. First, the 2D-COF exhibited low internode planarity, a marked VIS absorption associated with the perylene group, a pronounced porphyrin-to-perylene CT behavior, and strong thermal stability. Then, the few-layer models (2–3) revealed that the columnar growth of the COF layers is mainly governed by AA stacking interactions,^32^ within large interlayer distances (4 Å) due to the torsion of the benzene linkers rotating out of the porphyrin plane. However, when a fourth PDI-ZnP-COF layer is added, steric impediment results in a globally planar layered structure where COF layers are closely packed [i.e., interlayer distances (3.1 Å)]. Thus, the columnar growth led to pronounced interlayer orbital overlap, which translated to a band gap reduction, exchange of HOCO and LUCO positions on perylene and porphyrin fragments, respectively, thus interchanging the CT directionality toward the porphyrin, and to an absorption dominated by the porphyrin group.

To sum up, the results demonstrated the potential of stacking COFs combining functional building blocks to finely tune their structural and optoelectronic properties, thus being able to target multiple functions. For instance, it is possible to build high-performance photocatalytic devices by integrating PDI-ZnP-COFs with a small number of layers presenting more accessible active sites and acting as a photoactive wider bandgap semiconductor to generate charges, with thicker layered COFs displaying high conductivity in the stacking direction, which promotes the carrier migration and mitigates the device losses. Indeed, similar layer engineering approaches have already been developed to build multilayered inorganic structures combining both stability and efficiency.^37^ Therefore, the synergy between our theoretical outcomes and an experimental fine control of the growing process of multilayered high aromatic COFs will open the door to the practical applications of multifunctional highly efficient porous material-based optoelectronic devices.

## Supplementary Material



## Notes

Benchmarking of DFT-based methods, PDOS of the multilayered COFs estimated with the PBE functional, SEM micrography of PDI-ZnP COF, TEM micrographs of PDI-ZnP COF, calculated thermodynamic properties of the PDI-ZnP-COF monolayer, solid-state UV–vis spectrum of PDI-ZnP COF, excitation/emission spectra of PDI-ZnP COF, frontier crystalline orbitals of multilayered PDI-ZnP COFs, calculated excited state properties of PDI-ZnP COFs, occupied/virtual crystalline orbitals for the main excited states of PDI-ZnP-COFs, calculated IR ν­(N–C) frequency as a function of the number of 2D COF layers, and correlation of the HOCO-LUCO gaps computed with PBE and PBE0 functionals (PDF)
