# Supplementary material for: Stacking Effects on the Optoelectronic Properties of 2D Perylene-Zn-Porphyrin-Based COFs
Source: J Phys Chem C Nanomater Interfaces. 2026 Mar 10;130(12):4451–65. doi: 10.1021/acs.jpcc.5c08341 (PMC13034458; doi:10.1021/acs.jpcc.5c08341)
Supplement: Supplementary file 1 [file jp5c08341_si_001.pdf]

Supporting Information

Stacking Effects on the Opto-electronic Properties of 2D Perylene-Zn-porphyrin-based COFs

Valentin Diez-Cabanes[1,2], Sergio de-la-Huerta-Sainz,[1,2] Elisabeth Escamilla,[1] Pedro A. Marcos,[3] Alfredo Bol-Arreba,[1,3] Kathryn McCarthy,[4] Roberto González-Gómez,[4] Santiago Aparicio,[1,2] Pau Farràs[4*]*

[1] International Research Center in Critical Raw Materials for Advanced Industrial Technologies (ICCRAM), University of Burgos, 09001, Burgos, Spain

[2] Department of Chemistry, University of Burgos, 09001, Burgos, Spain

[3] Department of Physics, University of Burgos, 09001, Burgos, Spain

[4] School of Biological and Chemical Sciences, Ryan Institute, University of Galway, Galway, Ireland, H91 TK33

*Authors to whom correspondence should be addressed: sapar@ubu.es (S. Aparicio), pau.farras@universityofgalway.ie (P. Farràs)

Table S1. Comparison of energy gap values and frequency associated with the imide group for three calculation methods.

Monolayer PDI-ZnP-COF	LDA (eV)	GGA (eV)	DFT-TB (eV)
Energy gap (eV)	1.017	0.89	0.94
δ(N-C)$_{imide}$ (cm^{-1})	1119	1134	1125

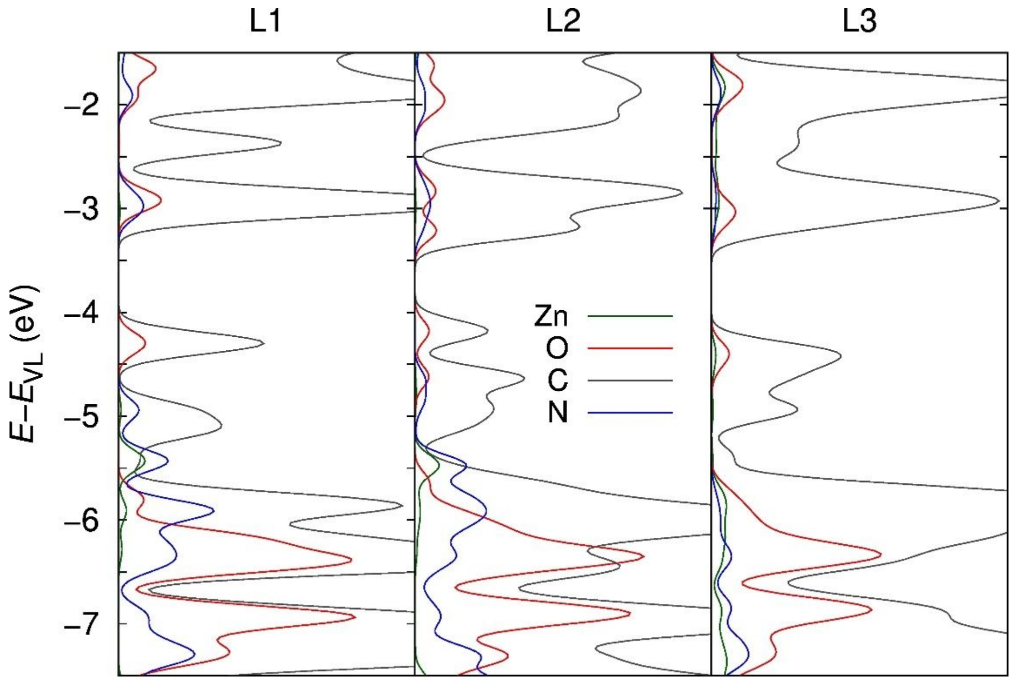

Figure S1. Projected density of states (PDOS) of the atoms conforming the PDI-ZnP 2D COFs owing one (L1), two (L2) and three (L3) layers, as calculated by employing pure DFT functionals (PBE), where the vacuum level energy (E_{VL}) was set as reference.

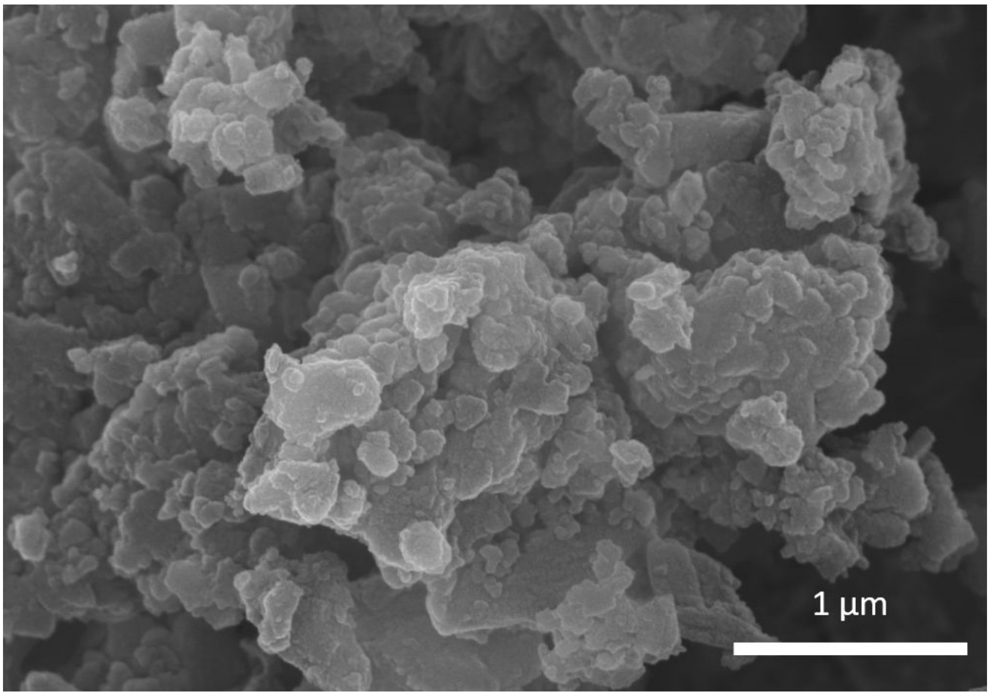

Figure S2. SEM micrography of PDI-ZnP COF.

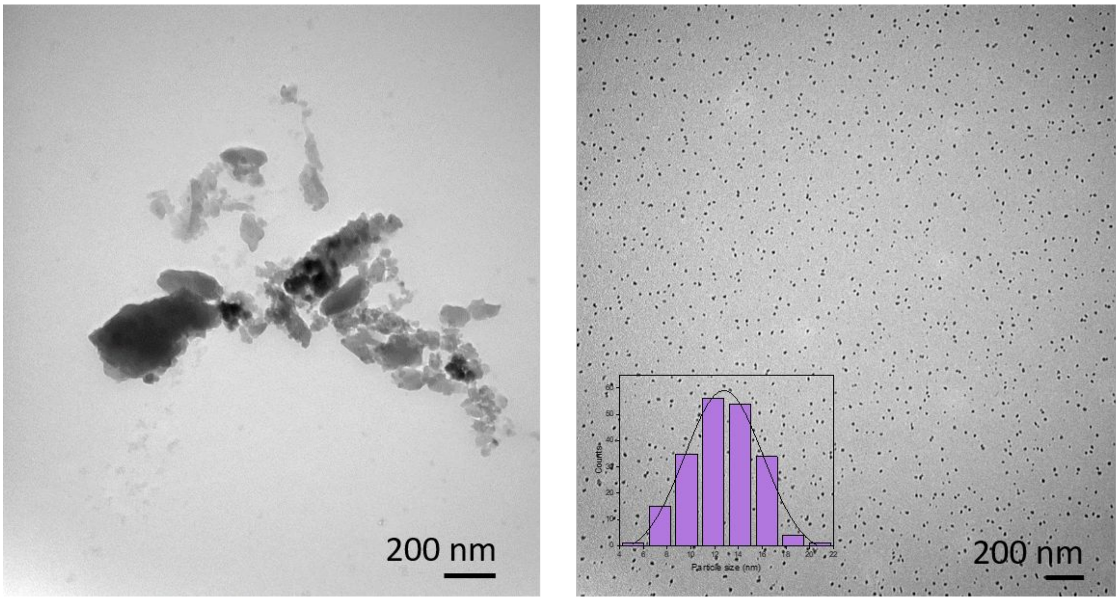

Figure S3. TEM micrographs of PDI-ZnP COF exfoliated in ethanol (left) and heptanoic acid (right). Size analysis distribution of nanospheres dispersed in heptanoic acid is presented in the inset.

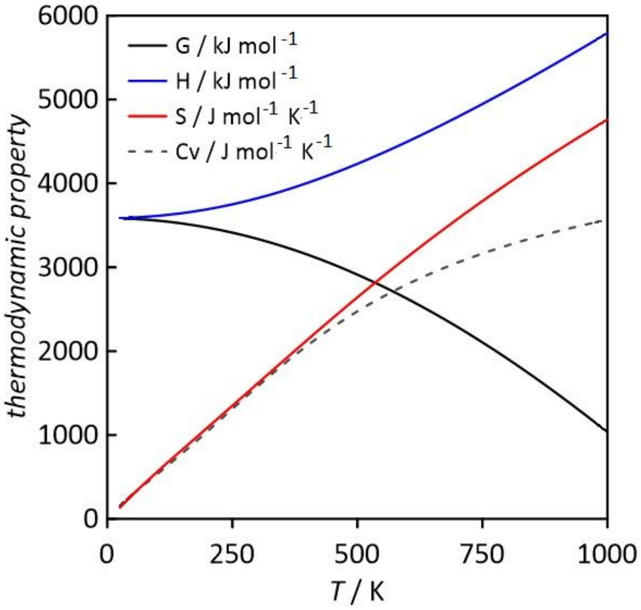

Figure S4. Calculated thermodynamic properties for the optimized structure of the considered 2D PDI-ZnP-COF monolayer.

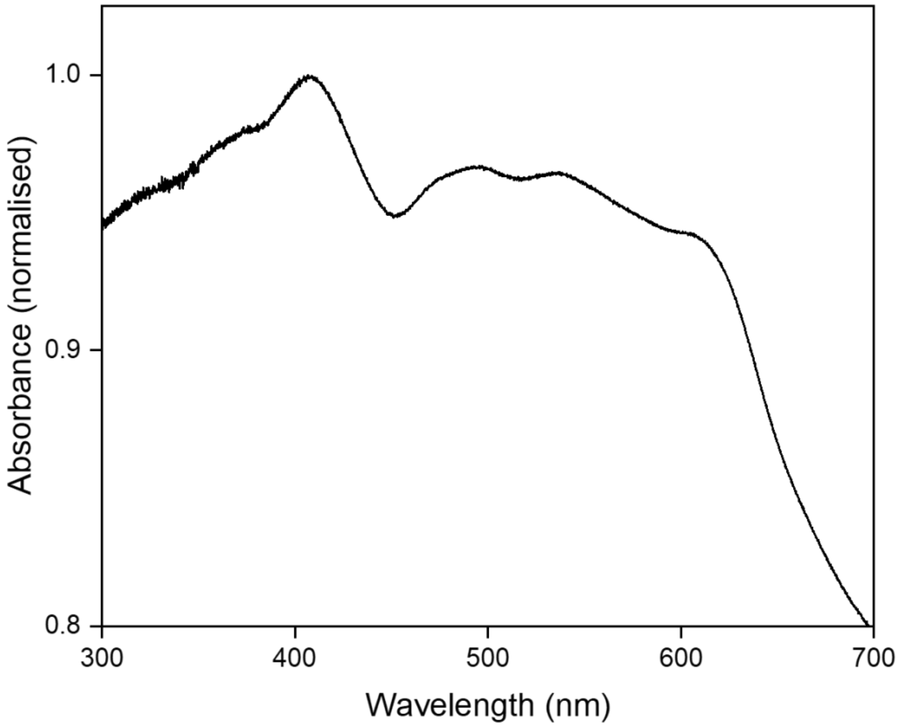

Figure S5. Solid-state UV-vis spectrum of PDI-ZnP COF.

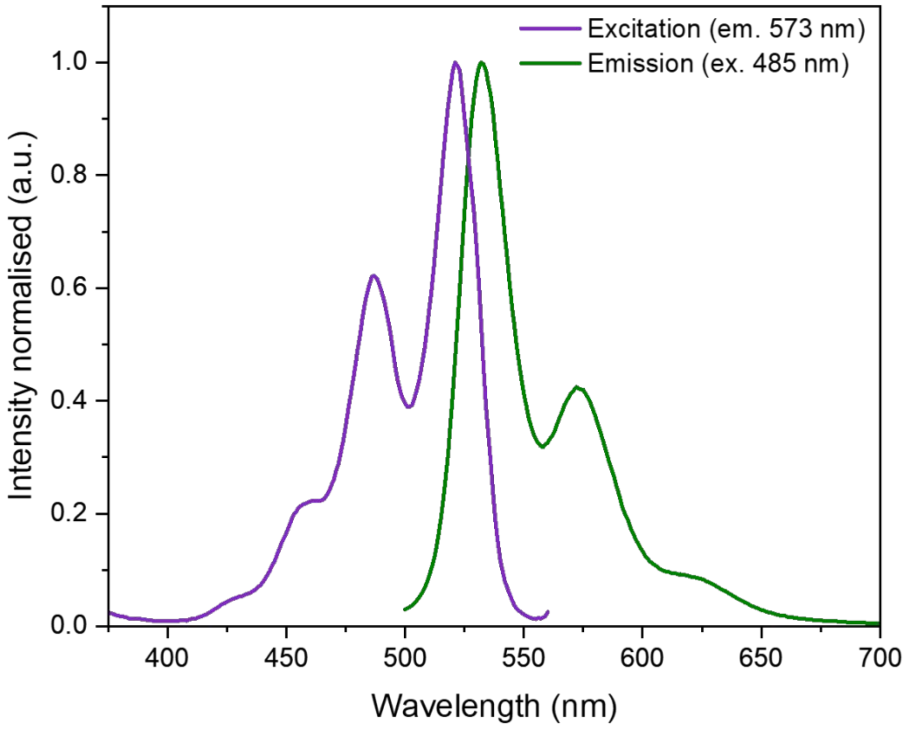

Figure S6. Excitation (purple) and emission (green) spectra of PDI-ZnP COF.

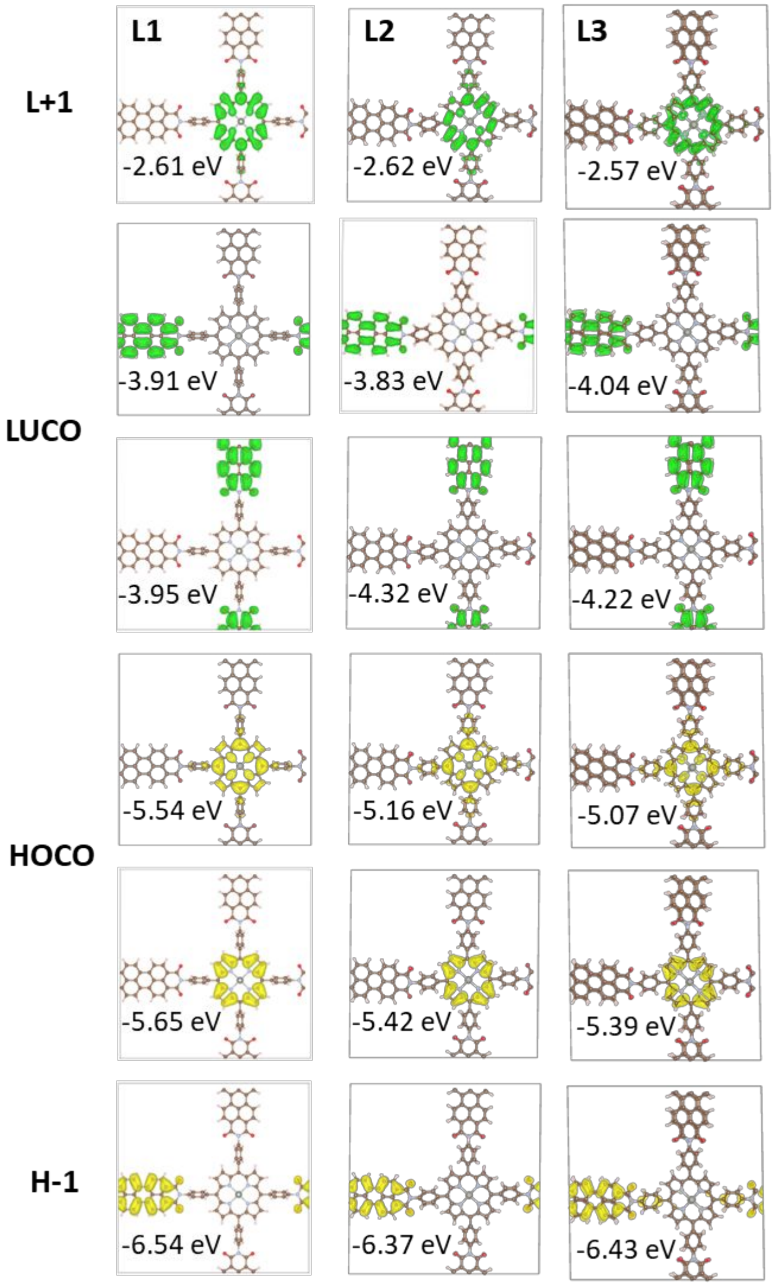

Figure S7. Top views of the shapes for the frontier crystalline orbitals indicated in the PDOS from Figure 4-a, for the 2D PDI-ZnP-COFs owing one (L1), two (L2) and three (L3) layers (from the left to the right). The iso-value used to plot the iso-surfaces was set to 0.02 a.u.

Table S2. Excited state characteristics of the main vertical transitions composing the absorption spectra of the PDI-ZnP 2D COFs owing one (L1), two (L2) and three (L3) layers: state number (*n*), exciton energy (E_x), oscillator strength (*f*) and main occupied→ virtual crystalline orbital transitions with their corresponding weight coefficients (C_i).

COF	*n*	E_x (eV)	*f*	occ→virt	C_i
L1	1	0.63	1.45E-05	H→L	-1.00
	2	0.66	1.16E-05	H→L+1	1.00
	31	2.09	4.92E-02	H-15→L	0.99
	51	2.37	1.88	H-4→L	0.91
	52	2.39	1.84	H-3→L+1	0.91
	158	3.32	0.539	H-10→L+7	0.50
	255	3.79	0.55	H→L+23	-0.62
L2	1	0.05	1.81E-05	H→L	0.80
	2	0.06	1.14E-05	H→L+1	-0.80
	66	1.48	1.22E-02	H-25→L+1	0.70
	174	2.09	0.503	H-3→L+9	0.60
	175	2.09	0.423	H-1→L+9	-0.57
L3	1	0.03	2.63E-03	H→L	1.00
	5	0.17	4.41E-03	H-2→L	1.00
	29	0.56	1.39E-03	H-3→L+5	1.00

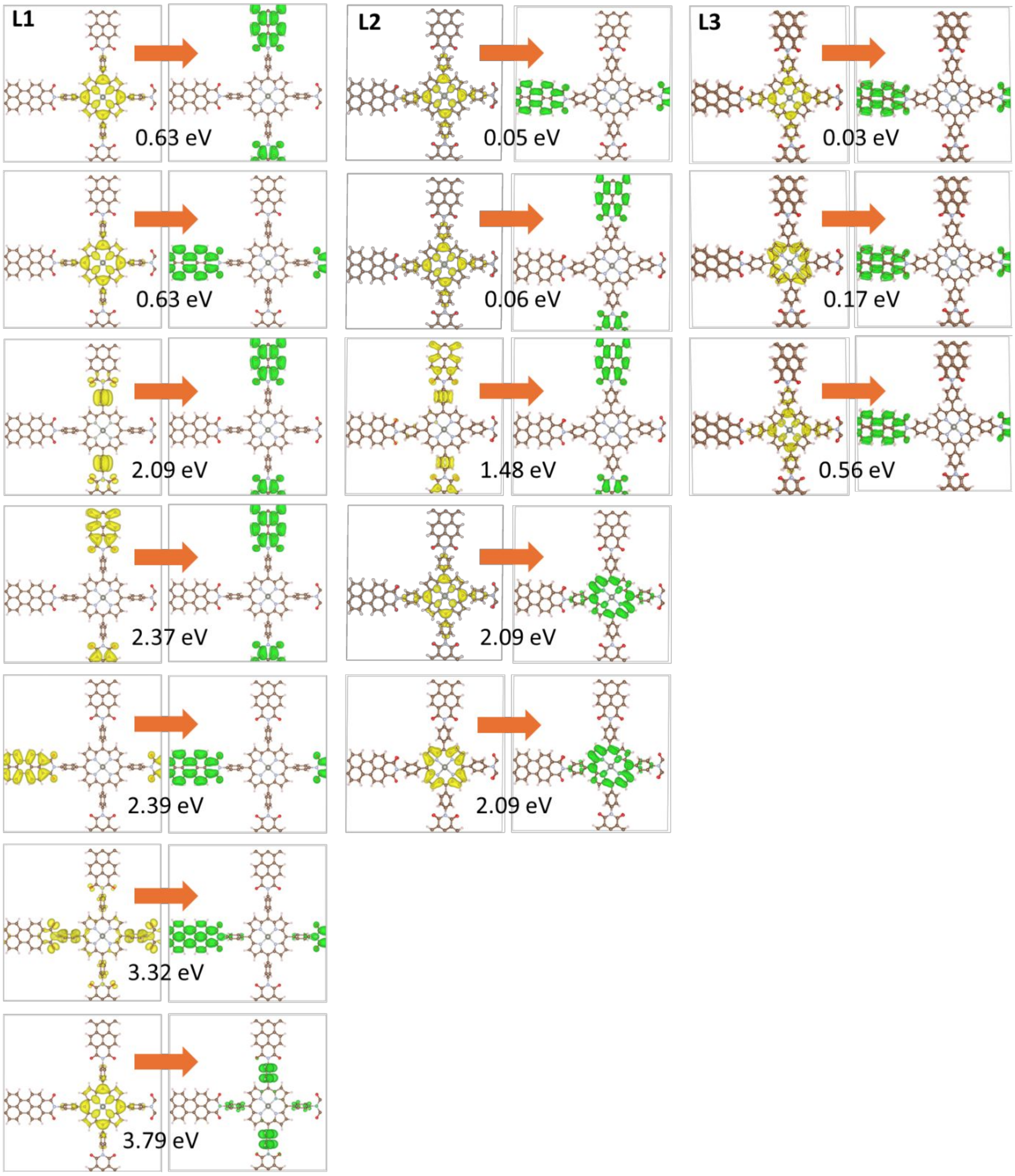

Figure S8. Top views of the shapes for the occupied (yellow) and virtual (green) crystalline orbitals involved in the main transitions of the excited states collected in Table S2 for the 2D PDI-ZnP-COFs owing one (L1), two (L2) and three (L3) layers (from the left to the right). The iso-value used to plot the iso-surfaces was set to 0.02 a.u.

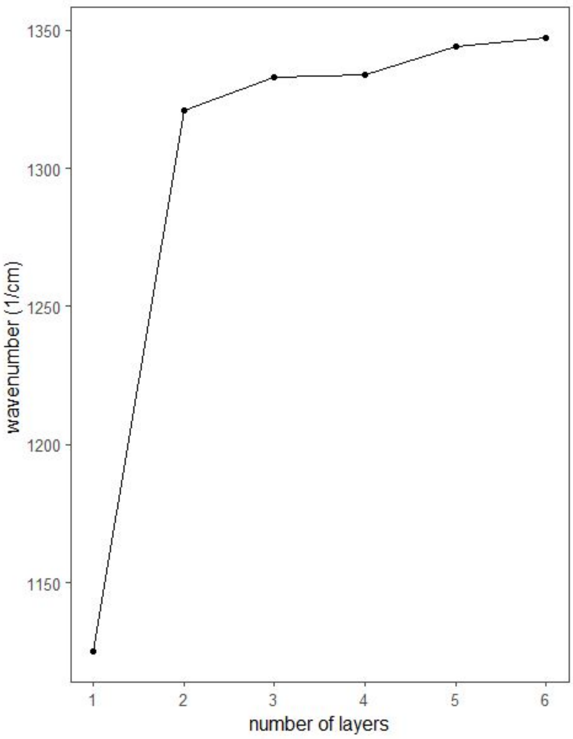

Figure S9. Calculated IR *v*(N-C) frequency as function of the number of layers (*n*=1-6) of the PDI-ZnP 2D COFs.

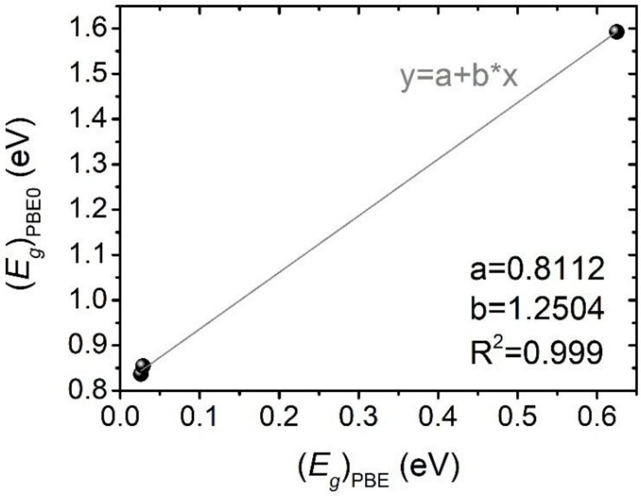

Figure S10. Correlation between the HOCO-LUCO gaps computed with pure (PBE, x-axis) and hybrid (PBE0, y-axis) DFT functionals, for the PDI-ZnP 2D COFs owing one (L1), two (L2) and three (L3) layers. The parameters from the linear fit have been recorded in set.
